# Development and Predictors of Sarcopenic Dysphagia during Hospitalization of Older Adults

**DOI:** 10.3390/nu12010070

**Published:** 2019-12-26

**Authors:** Keisuke Maeda, Yuria Ishida, Tomoyuki Nonogaki, Akio Shimizu, Yosuke Yamanaka, Remi Matsuyama, Ryoko Kato, Naoharu Mori

**Affiliations:** 1 Department of Palliative and Supportive Medicine, Graduate School of Medicine, Aichi Medical University, 1-1 Yazakokarimata, Nagakute, Aichi 480-1195, Japan; a.shimizu.diet@gmail.com (A.S.); nmori@aichi-med-u.ac.jp (N.M.); 2 Nutritional Therapy Support Center, Aichi Medical University Hospital, 1-1 Yazakokarimata, Nagakute, Aichi 480-1195, Japan; 3 Department of Nutrition, Aichi Medical University Hospital, 1-1 Yazakokarimata, Nagakute, Aichi 480-1195, Japan; okuda.yuria.785@mail.aichi-med-u.ac.jp; 4 Department of Pharmacy, Aichi Medical University Hospital, 1-1 Yazakokarimata, Nagakute, Aichi 480-1195, Japan; nonogaki.tomoyuki.562@mail.aichi-med-u.ac.jp (T.N.); inuduka.ryouko.907@mail.aichi-med-u.ac.jp (R.K.); 5 Department of Nutrition, Hamamatsu City Rehabilitation Hospital, Shizuoka 433-8127, Japan; 6 Department of Oral and Maxillofacial Surgery, Graduate School of Medicine, Aichi Medical University, 1-1 Yazakokarimata, Nagakute, Aichi 480-1195, Japan; yamanaka.yousuke.694@mail.aichi-med-u.ac.jp (Y.Y.); matsuyama.remi.721@mail.aichi-med-u.ac.jp (R.M.)

**Keywords:** acute care, bed rest, deconditioning, sarcopenia, swallowing difficulty

## Abstract

The study aimed to investigate the impact of sarcopenia and sarcopenia-related conditions on the development of swallowing disorders during hospitalization. Older adult inpatients (n = 8768) without swallowing disorders in the premorbid period were studied. Sarcopenia-related conditions were evaluated in terms of nutritional status, physical status, and ambulatory conditions as well as hand-grip strength and muscle mass assessed by calf circumference. Development of swallowing disorders was defined based on food texture at discharge from the hospital. The patients’ mean age was 76.1 ± 6.9 years. A total of 374 (4.3%) patients developed swallowing disorders during hospitalization. They were older, with poorer nutritional status, and had more decline of physical performance than those without swallowing disorders. Performance Status score (odds ratio (OR) = 1.28 (1.12–1.46) *p* < 0.001), ambulatory dependency (OR = 1.72 (1.09–2.71), *p* = 0.020), malnutrition score (OR = 0.92 (0.87–0.97), *p* = 0.002), insufficient nutritional intake (OR = 2.33 (1.60–3.40), *p* < 0.001), and length of stay (OR = 1.01 (1.00–1.01), *p* = 0.001) were independent contributing factors for swallowing disorder development in the multivariate analysis. The presence of possible sarcopenia was also a contributor to swallowing disorder development. In conclusion, swallowing disorders could develop in patients with possible sarcopenia and sarcopenia-related conditions during hospitalization. Clinicians should be aware of this risk and provide appropriate interventions to prevent sarcopenic dysphagia.

## 1. Introduction

Sarcopenia, a condition referring to the decline of both skeletal muscle mass and strength [1], impacts the health and quality of life of older adults. The onset and progression of sarcopenia depend on inactivity, undernutrition, and diseases as well as aging [2]. Recently, the association between sarcopenia and swallowing disorders has gained the attention of experts in the field of geriatric nutrition [3,4,5]. Four academic organizations in Japan specializing in geriatric nutrition and dysphagia rehabilitation published a consensus article focusing on the relationship between swallowing disorders and surrounding etiologies, the current definition and diagnosis of sarcopenic dysphagia, preventive and therapeutic strategies, and issues to be tackled regarding sarcopenic dysphagia [3]. The European Society for Swallowing Disorders is also concerned about sarcopenia and its influence on swallowing ability, especially in managing geriatric syndrome and nutrition in older adults [4]. Additionally, the first systematic review and meta-analysis reported an existing association between sarcopenia and swallowing disorders [5]. Previous studies found that muscle mass measured by anthropometry was positively correlated to muscle mass [6] and strength [7] of the tongue, and the chewing muscles’ strength was weaker in sarcopenic older adults than in non-sarcopenic ones [8]. Additionally, other studies have reported that decreased muscle mass [9] and strength [7,10] of the eating-related muscles were linked to swallowing disorders in older adults.

However, little is known about the causal relationship between sarcopenia and swallowing disorders. There may be a two-way causal relationship between them. The texture-modified diets consumed by patients with dysphagia [11], which contain a relatively low amount of energy and protein, and a sedentary lifestyle owing to disease- or aging-related disability along with swallowing disorders will cause nutrition- and activity-related sarcopenia, respectively. On the other hand, it can also be assumed that sarcopenia, occurring in the whole body, can affect the eating-related muscles, and the decreased muscle mass and strength of the muscles passively influence swallowing function [12]. A systematic review failed to screen studies investigating whether sarcopenia had caused the swallowing disorders or vice versa [5].

Hospitalization may accelerate the development of sarcopenic dysphagia, which is a swallowing disorder due to sarcopenia [13]. Several studies have reported that hospitalization reduces the muscle mass and strength of older inpatients [14,15], which could have resulted from insufficient nutrition, forced bedrest, and the presence of diseases causing sarcopenia during hospitalization. Some clinicians refer to the hospital-associated decline of muscle mass and strength as iatrogenic sarcopenia [16]. To make matters worse, the sarcopenia condition will further deteriorate in hospitalized patients. Excessively advanced sarcopenia during hospitalization will exacerbate swallowing disorders. Therefore, we hypothesized that older adults with sarcopenia or sarcopenia-related conditions such as aging, inactivity, undernutrition, and sarcopenia-related diseases at hospital admission are likely to develop swallowing disorders during hospitalization. The current study aimed to verify this hypothesis and to investigate the impact of sarcopenia and sarcopenia-related conditions on the frequency of swallowing disorders during hospitalization.

## 2. Materials and Methods

### 2.1. Participants

This retrospective, observational study examined consecutive patients aged ≥65 years who stayed for at least 4 days in a 900-bed academic hospital between December 2017 and November 2018. These patients had consumed regular texture food before the onset of the disease for which they were hospitalized. The exclusion criteria were patients who died in the hospital and those presented with diseases related to swallowing difficulties, including diseases of the nervous system (the International Classification of Diseases 10th Revision (ICD-10) category G), head and neck disease (ICD-10 category C00-14 and S00-19), and stroke (ICD-10 category I60-69) to eliminate the influence of these diseases on swallowing ability. The study was approved by the ethics committee of the hospital (approval ID: 2019-H031). Given that this study was retrospective in nature, we could not obtain written informed consent prior to the study; therefore, the ethics committee waived the requirement for written informed consent. Additionally, an opt-out procedure was conducted to give all patients an opportunity to remove themselves from the study by informing the patients of the study protocol on the hospital homepage, as advised by the committee.

### 2.2. Texture of Food and Outcome

All patients or their guardians were interviewed by trained nurses on the admission day regarding daily, usual, and premorbid texture of food. Usually, nurses asked whether patients eat normal, soft, minced, pureed, or liquidized rice as the main source of carbohydrates, and normal, soft, minced, pureed, or liquidized for other foods. In the study, normal and soft rice was considered as a regular texture of food, because dysphagic patients would usually be considered to consume minced, pureed, or liquidized food [17].

The primary outcome of the study was whether patients consumed a texture-modified diet or were placed on a nil by mouth status at hospital discharge. Patients who did not eat food with regular texture were considered to have a decline in swallowing ability, since the study included only patients consuming food with regular texture during the period of premorbid daily living. We collected information about food and nutrition delivered on the final day of hospitalization by reviewing the medical charts. Patients who were provided minced, pureed, or liquidized food or were placed on nil by mouth status, indicated as Functional Oral Intake Scale ≤5 [18], were considered to have a decline in swallowing ability during the hospitalization period.

### 2.3. Disease, Nutritional, and Physical Status

Diseases leading to the hospitalization and comorbidities were collected from ICD-10 records in the medical charts. To be useful, these diseases were classified into ICD-10-defined major categories. Furthermore, information of comorbidities was evaluated using the Charlson Comorbidity Index (CCI) [19]. Nutritional variables including body mass index (BMI), the Mini Nutritional Assessment Short Form (MNA-SF) [20], and amount of food intake at admission were recorded. BMI was calculated using the following equation: BMI = actual body weight (kg)/squared body height (m^2^). MNA-SF, which was assessed by trained nurses, consisted of an ordinal scale with values ranging from 0 to 14 points after evaluating six subitems. Scores of 0–7, 8–11, and 12–14 represent malnourished, at risk of malnutrition, and normal nutritional status, respectively. Food intake information obtained by interviewing patients/guardians at the time of admission was categorized into sufficient, insufficient, or none for 5 days just before admission. Overall physical condition using the Eastern Cooperative Oncology Group defined Performance Status (PS) was evaluated. Premorbid mobility was assessed using three categories: (1) patient goes out, (2) able to get out of bed/chair but does not go out, and (3) bed/chair bound.

### 2.4. Sarcopenia

The subgroup analyses included consecutive patients who had undergone nutritional assessment by nutrition support teams working in the hospital. The nutritional support teams usually perform nutritional assessment in patients revealed to have a nutritional risk based on a validated screening tool, such as the MNA-SF. The nutritional support teams evaluate sarcopenia as a standard procedure of nutritional assessment. Skeletal muscle mass was evaluated based on calf circumference, and muscle strength was evaluated based on hand-grip strength. Given that the study was performed in clinical practice, we considered it useful to detect decreased skeletal muscle mass using simple and noninvasive examinations, such as calf circumference, which is known to be acceptable in evaluating decreased muscle mass during nutritional assessment in clinical practice [21], obtained through bedside assessment in this relatively large sample study. Calf circumference ≤30 cm for male and ≤29 cm for female Asian and hospitalized older adults was employed to detect decreased skeletal muscle mass in the study [22]. Moreover, hand-grip strength <26 kg for men and <18 kg for women was employed to detect a decline in muscle strength according to the Asian Working Group of Sarcopenia criteria [23]. Patients who presented with both decreased muscle mass and a decline in muscle strength were diagnosed as having sarcopenia.

### 2.5. Statistical Analyses

Categorical variables are expressed as the number of patients (percentage). Quantitative variables, including parametric and nonparametric values evaluated on histogram, are expressed as the mean ± standard deviation and median (interquartile range), respectively. Comparisons between groups were made using the chi-squared test, Student’s *t*-test, or Mann–Whitney U-test for categorical, parametric, or nonparametric variables, respectively. Frequency of swallowing difficulty at discharge is expressed in graphs with obtained frequency and estimated 95% confidence intervals (CI). A multivariate logistic regression analysis was performed to identify association between baseline variables and swallowing difficulty at discharge. Length of hospital stay was also adjusted in the multivariate analysis. Covariates adjusted in the multivariable analyses were determined using a directed acyclic graph, resulting in the following covariates: age, sex, CCI, PS, mobility, MNA-SF, amount of food intake, and primary disease for admission. Additionally, another multivariate logistic regression analysis was performed to identify the relationship between sarcopenia and swallowing difficulty at discharge in the subgroup analysis. We used logit propensity score for sarcopenia calculated from all other baseline variables to reduce the variables in the model because of the relatively small number of patients with swallowing difficulty at discharge in the subgroup population. *p*-values < 0.05 were considered statistically significant. Statistical analyses were performed using SPSS 23.0 software (IBM Japan, Tokyo, Japan).

## 3. Results

The study examined the eligibility of 10,204 patients who fulfilled the inclusion criteria during the study period. A total of 1436 patients were excluded due to mortality (355 cases, 3.5%), disease of the nervous system (260 cases, 2.5%), head and neck disease (126 cases, 1.2%), and stroke (695 cases, 6.8%). Finally, 8768 patients with a mean age of 76.1 years were analyzed. The proportion of male patients was 55.1%. Based on the ICD-10 codes for the reason for hospital admission, neoplasms (30.5%), circulatory diseases (15.0%), digestive diseases (12.5%), and respiratory diseases (8.9%) were the major causes for admission.

**Figure 1:**
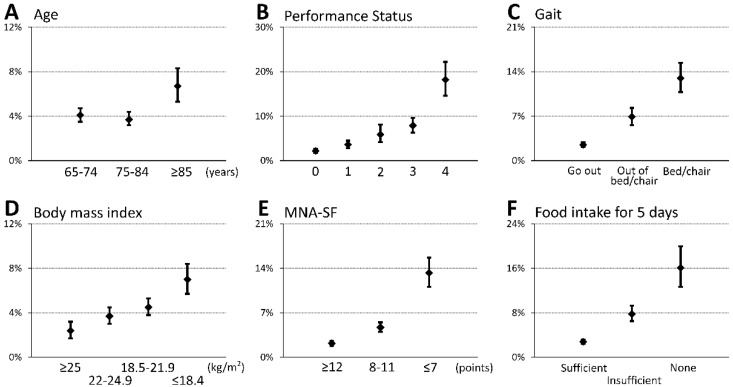
Prevalence of swallowing disorders at discharge. Percentages and 95% confidence interval of patients with swallowing disorders at the time of discharge from hospital in the groups stratified by age (**A**), Performance Status (**B**), mobility status (**C**), body mass index (**D**), nutritional status assessed by MNA-SF (**E**), and food intake at admission (**F**). The worse the condition observed at admission deteriorates, the more patients suffer from swallowing disorders during hospitalization. Abbreviations: MNA-SF, Mini Nutritional Assessment Short Form.

**Figure 2:**
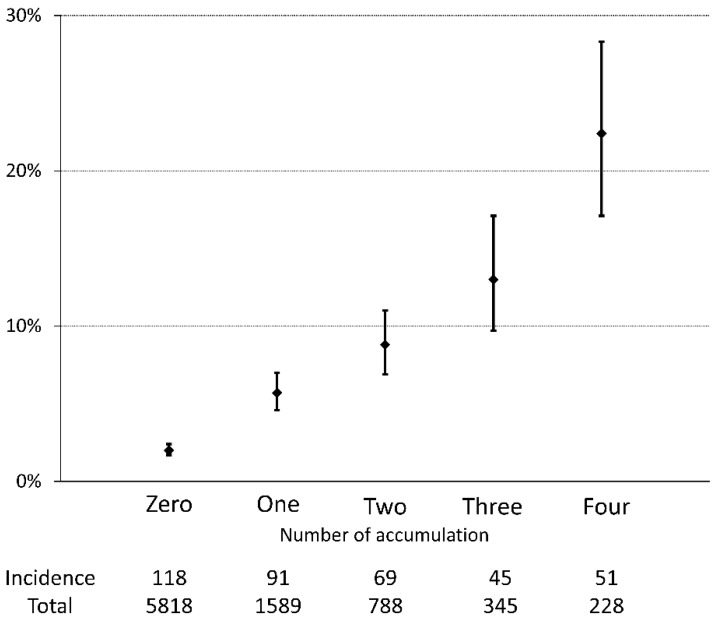
Prevalence of swallowing disorders and number of accumulation of impaired conditions. Percentages and 95% confidence interval of patients with swallowing disorders at the time of discharge from hospital were depicted according to number of accumulation of the presence of sarcopenia-related conditions such as Performance Status 3/4, mobility status of bed/chair, MNA-SF ≤7, and insufficient/none food intake. All comparisons showed a statistical significant difference, except for two vs. one and three after Bonferroni correction. Abbreviations: MNA-SF, Mini Nutritional Assessment Short Form.

A total of 374 (4.3%) patients had swallowing disorders at discharge in this cohort (Table 1). The patients with dysphagia at discharge were likely to be older (*p* = 0.011), have a lower BMI (*p* < 0.001) and MNA-SF score (*p* < 0.001), have more comorbidities (CCI, 3 (1–8) vs. 2 (1–4), *p* < 0.001), be inactive (PS, *p* < 0.001; mobility, *p* < 0.001), and have poorer food intake (*p* < 0.001). Additionally, patients with dysphagia were hospitalized longer than patients without dysphagia (29.2 ± 21.6 vs. 20.0 ± 8.8 days, *p* < 0.001). Figure 1 shows that the incidence of swallowing disorder was more frequently seen in patients with older age, poor PS, ambulation difficulty, low body weight, malnutrition, and poorer food intake. Multivariate analysis shows that inactive status assessed by PS, sedentary condition, poor nutritional status by MNA-SF, and poor food intake were independent contributors of decline in swallowing ability during hospitalization (Table 2). Furthermore, along with the increased number of impaired conditions, incidences of swallowing disorders were more frequently observed when counting such impaired conditions (Figure 2). The frequency of patients with low physical activity (PS 3/4) also increased when counting such impaired conditions (0.0%, 26.3%, 67.8%, 97.4%, and 100% in those with 0, 1, 2, 3, and 4 impaired conditions, respectively, *p* < 0.001). The predictive probability was 10 times higher in patients with low PS and sedentary, poor nutrition, and poor intake conditions than in those without such conditions (22.4% vs. 2.0%, *p* < 0.001).

**Figure 3:**
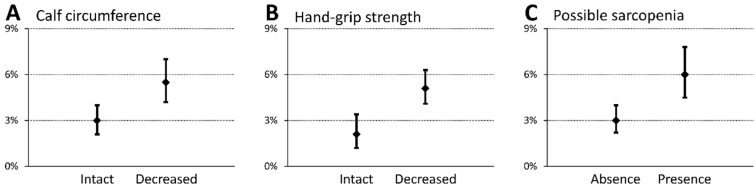
Prevalence of swallowing disorders regarding possible sarcopenia indicators in a subgroup analysis. Percentages and 95% confidence interval of patients with swallowing disorders at the time of discharge from hospital with comparisons between groups in terms of calf circumference (**A**), hand grip strength (**B**), and possible sarcopenia (**C**). Patients presenting with decreased muscle mass and strength and possible sarcopenia at admission are likely to develop swallowing disorders during hospitalization.

The subgroup analysis included 2384 patients who underwent nutritional assessment owing to nutritional risk screening results. Of these, 98 (4.1%) patients developed swallowing disorders (Table 3). They had lower hand-grip strength and a smaller calf circumference than those who did not develop swallowing disorders, showing a statistically significant difference. Moreover, possible sarcopenia at admission was more prevalent in patients who developed swallowing disorders than in those who did not (55.1% vs. 37.0%, *p* < 0.001). Figure 3 shows the incidence of swallowing disorders during hospitalization. Patients with sarcopenia at baseline had swallowing ability deterioration and decreased muscle mass and strength. Finally, the multivariate analysis adjusted for possible confounders detected that sarcopenia was an independent predictor for swallowing disorders at the time of discharge (odds ratio 1.622 (1.028–2.559), *p* = 0.038) (Table 4).

## 4. Discussion

This study was conducted to investigate whether sarcopenia or sarcopenia-related conditions had an influence on the development of swallowing disorders during hospitalization in older adult patients in an academic hospital. The subjects without swallowing disorders prior to the onset of the admission disease were examined in a large cohort. We found two novel findings regarding sarcopenic dysphagia. First, sarcopenia and sarcopenia-related conditions were possible contributors to the development of swallowing disorders in older inpatients, which support our hypothesis. Second, the higher the number of impaired conditions accumulated, the more frequent the occurrence of swallowing disorders.

Activity- and nutrition-related unfavorable conditions were independently associated with swallowing disorder development, and in the subgroup analysis, the simplified diagnosis of sarcopenia also predicted the development of such a condition. The results indicated that hospital-associated dysphagia, referring to a swallowing disorder acquired during hospitalization [13], mostly seemed to be sarcopenic dysphagia. A previous study investigating older inpatients with were prohibited oral ingestion at admission to the hospital reported that only patients with sarcopenia developed swallowing disorders within two months, and lower muscle mass and poorer physical function were the predictors of sarcopenic dysphagia [12]. The development of swallowing disorders was considered to be caused by the further deterioration of activity- and nutrition-related conditions during hospitalization [24] in patients with sarcopenia. Both previous and current studies excluded some diseases, such as stroke, neurological disease, and head and neck diseases, commonly known as causal diseases of swallowing disorders, to eliminate disease-related influences, and these reported similar conclusions. To the best of our knowledge, there are few studies reporting hospital-associated dysphagia rather than dysphagia due to such common diseases. We found that older adult patients with sarcopenia and conditions related to sarcopenia were exposed to hazards of hospital-associated dysphagia including sarcopenic dysphagia. Swallowing-related muscles differ embryologically from the skeletal muscles of the extremities and the trunk [3]. Swallowing-related muscles are not expected to develop dysfunction as they play a vital role in maintaining optimal nutrition status, similar to respiratory muscles, which are important for maintaining proper respiration. However, excessive progression of decreased muscle mass and strength will affect these functions, even in such dominant muscles. Therefore, existing deconditioning at admission would be an independent contributor for swallowing disorder development because the deconditioning might become much worse. The results demonstrated that the length of hospital stay was also associated with the development of dysphagia. This may indirectly explain the progression of deconditioning during hospitalization, and hospital-associated dysphagia would be probably considered to be hospital-acquired dysphagia.

It is controversial whether hospitalization would always worsen physical function in older adults. A systematic review reported that acute admission, but not elective admission, did not decrease muscle mass and strength [14]. Additionally, Van Ancum et al. reported that, in a study involving approximately 85% acute admission cases, muscle strength increased during hospitalization [25]. An acute condition may result in the underestimation of muscle strength due to acute symptoms such as fatigue, decreased temperature, pain, and so on. Although the current study did not examine whether the subjects were admitted owing to an emergent situation, the majority of patients admitted for cancer treatment might be primarily elective admissions. There may be characteristics of patients whose muscle mass and strength tend to be exacerbated. New incidence of sarcopenia among older adult inpatients occurred more often in patients with much older age, nutritional risk, such as lower body weight, and inactivity such as lower activities of daily living and longer bedrest duration than in those without such conditions [15]. These conditions could be regarded as age-, nutrition-, and activity-related conditions, which contribute to sarcopenia progression [2]. Given that patients with sarcopenia would have one or more of these conditions, we considered that sarcopenia would be the characteristic predicting exacerbation of muscle mass and strength.

We found that patients with a cumulative number of activity- and nutrition-related conditions at baseline were likely to develop a swallowing disorder during hospitalization. The predictive probability of patients with all four conditions showed 10 times higher risk of developing swallowing disorders than patients without such conditions. Accumulation of geriatric deconditioning would reflect progression of sarcopenia. A prospective study including older adult inpatients reported that the cumulative number of unfavorable geriatric conditions, such as risk of fall, delirium, malnutrition, and dependency, was negatively correlated to the amount of muscle mass and degree of muscle strength [25]. The results of our current study might signify a similar conclusion that patients with deconditioning accumulation had greater sarcopenia progression than those without accumulation, even in patients with sarcopenia. We evaluated PS, ambulatory status, nutritional status, and amount of oral intake in the study. While it was fortuitous, the number of these items showing deconditioning correlated to the frequency of swallowing disorders. These four items can be applied in clinical practice to screen patients with a potential risk of sarcopenic dysphagia because the items can be assessed readily without cost and invasive procedures. Other candidates for predicting sarcopenic dysphagia should be identified by further research studies.

We found that the frequency of patients with low physical activity (PS 3/4) also increased when counting sarcopenia-related conditions that were related to an increased incidence of dysphagia. Inactivity during hospitalization might affect the progression of decreased muscle mass and function. Generally, physical exercise is a powerful stimulus to attenuate physiological skeletal muscle alterations [26]. Physical exercise should be provided to patients with low physical activity in acute care hospitals. However, specific training programs to prevent the development of sarcopenia and further deterioration of muscle mass and function during hospitalization have not been clarified. A novel rehabilitation approach including an individualized physical training program to prevent the development of sarcopenic dysphagia in hospitalized patients should be created.

There are some limitations of the study. First, the study did not fully eliminate disease-induced swallowing disorders, although common diseases that can cause the condition such as stroke and diseases of central nervous system, head, and neck were excluded. Additionally, the influence of disease was not examined, although the information of disease was adjusted in the multivariate analyses. The influence should be investigated by separate clinical research for each disease. Second, we used the simplified criteria to diagnose sarcopenia for the specified group. It is recommended to use imaging devices or other invasive measurement to diagnose sarcopenia; however, it is difficult to perform this examination in large-size studies involving hospitalized patients. Third, the lack of standardized terminology for food consistency and fluid density is a limitation of this retrospective study, and thus, an exact diagnosis of the decline in swallowing ability may be difficult. Mucositis or esophagitis could develop when changing the food texture, although these diseases do not often cause dysphagia. Dysphagia is diagnosed based on not only food texture but also oral, transit, pharyngeal, and esophageal problems because the swallowing process is very complex. Moreover, multiple conditions (e.g., infections, acute organ failure) that may develop during hospitalization may cause swallowing disorders. Our study did not examine the onset of such conditions during hospitalization and their impact on the swallowing ability.

## 5. Conclusions

The current study provided evidence that swallowing disorders could develop in older patients with sarcopenia and sarcopenia-related conditions, and the swallowing disorders are more accurately termed sarcopenic dysphagia. The accumulation of sarcopenia-related conditions decuples the risk of sarcopenic dysphagia during hospitalization. Clinicians should be aware of this risk and provide appropriate interventions to prevent the development of sarcopenic dysphagia in this patient cohort.

## Figures and Tables

**Table 1:** Baseline characteristics and oral intake status at discharge.

	All	Regular Diet	TMD/NBM	*p*
	*n* = 8768	*n* = 8394	*n* = 374	
Age, years	76.1 ± 6.9	76.1 ± 6.9	77.0 ± 8.1	0.011
Male, *n* (%)	4831 (55.1)	4612 (54.9)	219 (58.6)	0.184
BMI, kg/m^2^	22.0 ± 3.7	22.0 ± 3.7	20.8 ± 3.8	<0.001
MNA-SF, score	11.0 ± 2.5	11.1 ± 2.4	9.1 ± 3.0	<0.001
Normal, *n* (%)	4439 (50.6)	4343 (51.7)	96 (25.7)	<0.001
At risk, *n* (%)	3471 (39.6)	3307 (39.4)	164 (43.9)	
Malnutrition, *n* (%)	858 (9.8)	744 (8.9)	114 (30.5)	
CCI, score	2 (1–4)	2 (1–4)	3 (1–8)	<0.001
PS, *n* (%)				
0	4598 (52.4)	4496 (53.6)	102 (27.3)	<0.001
1	2046 (23.3)	1973 (23.5)	73 (19.5)	
2	608 (6.9)	572 (6.8)	36 (9.6)	
3	1093 (12.5)	1007 (12.0)	86 (23.0)	
4	423 (4.8)	346 (4.1)	77 (20.6)	
Mobility, *n* (%)				
Goes out	6440 (73.4)	6277 (74.8)	163 (43.6)	<0.001
Able to get out of bed/chair, but does not go out	1488 (17.0)	1386 (16.5)	102 (27.3)	
Bed/chair bound	840 (9.6)	731 (8.7)	109 (29.1)	
Food intake, *n* (%)				
Sufficient	6870 (78.4)	6678 (79.6)	192 (51.3)	<0.001
Insufficient	1488 (17.0)	1372 (16.3)	116 (31.0)	
None	410 (4.7)	344 (4.1)	66 (17.6)	

Abbreviations: n, number; TMD, texture modified diet; NBM, nil by mouth; BMI, body mass index; MNA-SF, Mini Nutritional Assessment Short Form; CCI, Charlson Comorbidity Index; PS, Eastern Cooperative Oncology Group-defined Performance Status.

**Table 2:** Multivariate logistic regression analysis for swallowing problems.

Variables	Adjusted OR	95% CI	*p*
Age	0.994	0.978–1.010	0.461
Male sex	1.148	0.921–1.429	0.219
CCI score	1.017	0.983–1.053	0.329
PS score	1.278	1.119–1.459	<0.001
Gait (reference: goes out)			
Out of bed/chair	1.249	0.879–1.775	0.215
Bed/chair	1.716	1.088–2.707	0.020
MNA-SF score	0.919	0.871–0.970	0.002
Food intake (reference: sufficient)			
Insufficient	1.586	1.205–2.088	0.001
None	2.333	1.602–3.397	<0.001
Length of hospital stay	1.008	1.003–1.013	0.001
Primary disease for admission	abbreviated		

Abbreviations: OR, odds ratio; CI, confidence interval; CCI, Charlson Comorbidity Index; PS, Eastern Cooperative Oncology Group-defined Performance Status; MNA-SF, Mini Nutritional Assessment Short Form.

**Table 3:** Subgroup analysis regarding sarcopenia indexes.

	All	Regular Diet	TMD/NBM	*p*
	*n* = 2384	*n* = 2286	*n* = 98	
Age, years	77.9 ± 7.1	77.9 ± 7.0	78.6 ± 8.8	0.340
Male sex, *n* (%)	1285 (53.9)	1223 (53.5)	62 (63.3)	0.063
Hand-grip strength, kg				
Men	24.5 ± 8.5	24.7 ± 8.4	21.3 ± 8.6	0.002
Women	14.7 ± 5.6	14.7 ± 5.6	12.8 ± 5.6	0.043
Calf circumference, cm				
Men	30.9 ± 3.4	31.0 ± 3.4	29.8 ± 3.9	0.012
Women	29.3 ± 3.3	29.4 ± 3.2	28.0 ± 3.5	0.012
Possible sarcopenia, *n* (%)	900 (37.8)	846 (37.0)	54 (55.1)	<0.001

Abbreviations: TMD, texture modified diet; NBM, nil by mouth; n, number.

**Table 4:** Multivariate logistic regression analysis of a subgroup.

Variables	Adjusted OR	95% CI	*p*
Age	0.990	0.954–1.029	0.604
Male	1.466	0.958–2.244	0.078
PS score	1.191	0.934–1.518	0.159
Gait (reference: goes out)			
Out of bed/chair	1.353	0.732–2.502	0.335
Bed/chair	1.782	0.778–4.080	0.172
MNA-SF score	0.981	0.857–1.124	0.786
Food intake (reference: sufficient)			
Insufficient	1.974	1.220–3.194	0.006
None	2.136	1.019–4.481	0.045
Possible sarcopenia	1.622	1.028–2.559	0.038

Abbreviations: OR, odds ratio; CI, confidence interval; PS, Eastern Cooperative Oncology Group-defined Performance Status; MNA-SF, Mini Nutritional Assessment Short Form.
